# Changes in food habits amongst Norwegian adolescents in 2016 and 2019 according to gender and socioeconomic status

**DOI:** 10.29219/fnr.v65.6262

**Published:** 2021-12-30

**Authors:** Tonje H. Stea, Kristin Holvik, Caroline S. Bryntesen, Janicke B. Myhre

**Affiliations:** 1 Department of Health and Nursing Science, University of Agder, Kristiansand, Norway; 2 Department of Child and Adolescence Mental Health, Sørlandet Hospital, Kristiansand, Norway; 3 Department of Chronic Diseases and Ageing, Norwegian Institute of Public Health, Oslo, Norway; 4 Department of Nutrition, University of Oslo, Oslo, Norway

**Keywords:** adolescents, dietary habits, questionnaires, repeated cross-sectional studies, socio-demographic factors, time trends

## Abstract

**Background:**

Monitoring dietary habits is important in order to identify risk groups and as a basis for targeted public health initiatives.

**Objective:**

To study trends in consumption of selected foods and beverages from 2016 to 2019 amongst Norwegian adolescents according to gender and parental education.

**Design:**

Repeated cross-sectional study amongst 25,996 adolescents, aged 14–17 years old. Consumption of selected food and beverages was measured by an online food frequency questionnaire and general linear models were applied to estimate changes in dietary habits.

**Results:**

Between 2016 and 2019, we observed a reduced frequency of consumption of vegetables (from 4.7 to 4.4 times/week), fruit and berries (from 4.4 to 4.2 times/week), whole-grain bread (from 5.1 to 4.2 times/week), and fish (from 2.3 to 1.6 times/week). During this time period, we also observed a reduced frequency of consumption of salty snacks (from 2.1 to 1.9 times/week), sweets (from 2.3 to 2.0 times/week), sugar-sweetened beverages (from 2.8 to 2.6 times/week), and artificially sweetened beverages (from 2.2 to 1.5 times/week). In girls, there was a decrease in the reported frequency of consumption of fruit and berries (−4%, vs. no change in boys). The decrease in consumption frequency of whole-grain bread was larger in girls than in boys (−19% vs. −14%). Further, a 17% decrease in consumption of sweets was observed amongst adolescents with no or only one parent having college/university education compared to a 13% decrease in adolescents whose both parents had college/university education.

**Conclusion:**

Our results showed a decrease in frequency of consumption of selected healthy and unhealthy food and beverages amongst adolescents between 2016 and 2019. The gender gap in consumption of fruit and berries and whole-grain bread seemed to decrease during this time period, and the socio-economic gap in consumption of sweets seemed to disappear.

## Popular scientific summary

- Between 2016 and 2019, a reduced frequency of consumption of selected healthy and unhealthy food and beverages was observed amongst Norwegian adolescents.
- During this time period, the gender gap in consumption of fruit, berries and whole-grain bread seemed to decrease and the socioeconomic gap in consumption of sweets seemed to disappear.
- Future efforts should specifically focus on increasing the consumption of vegetables, fruit, berries, whole-grain bread and fish, and develop targeted intervention strategies to further decrease the gender and socio-economic gap in dietary habits.

A healthy diet is important for the prevention of chronic diseases. In 2017, the Global Burden of Disease (GBD) framework estimated that 11 million deaths and 255 million disability-adjusted life-years (DALYs) could be attributed to dietary risk factors, and that cardiovascular disease, stroke, and type 2 diabetes were the leading causes of diet-related deaths and DALYs (1, 2). Beneficial effects of the improved diet quality on health and well-being have been widely acknowledged (3). On the other hand, poor dietary habits characterised by high levels of sugar-sweetened beverages and sodium, and low levels of whole grains, fruit, vegetables, seafood omega 3 fatty acids, fibre, polyunsaturated fatty acids, legumes have been identified as major risk factors of deaths and DALYs in the GBD framework (4, 5).

As results from previous studies have indicated that dietary habits developed in childhood and adolescence may persist into adulthood (6–8), promoting healthy dietary habits from an early age is important (9, 10).

European studies have shown that a majority of adolescents consume more fat, sugar-sweetened beverages, and sweets, and less than half the amount of fruit and vegetables recommended by the World Health Organization (11–13). In a nationwide study from Norway, the intake of saturated fat and added sugar was higher, and the consumption of fruit, vegetables, and fish was lower than national dietary recommendations (14).

A systematic assessment of gender differences in diet quality in 187 countries has shown that women tend to have an overall healthier diet characterised by more fruit and vegetables, whole grains, and fish compared to men (15). Furthermore, studies amongst Norwegian adolescents have indicated a higher consumption of fruit and vegetables and a lower consumption of sugar-sweetened beverages amongst girls, but no gender differences in consumption of sweets (16, 17).

A social gradient in dietary habits has also been identified across the lifespan both in Norway and internationally (14, 17–20). Amongst adolescents with low socioeconomic status (SES), studies have suggested a lower consumption of healthy food items such as fruit, vegetables, and fish, and higher consumption of unhealthy food items and beverages such as sugar-sweetened beverages and sweets compared to those with high SES (14, 16, 17, 21, 22).

Reducing social inequalities in health and life expectancy is a goal of public health initiatives in many countries. In 2017, the Norwegian Government prepared a National Action Plan for a Healthier Diet, with an overarching goal of reducing socioeconomic differences in dietary habits and, specifically, increasing the consumption of fruit and vegetables, fish, and whole grains, and reducing the consumption of added sugar, saturated fat, and salt (23).

Thus, the aim of the present study was to examine changes in frequency of consumption of selected healthy foods (vegetables, fruit and berries, whole-grain bread, fish) and unhealthy foods and beverages (salty snacks, sweets, sugar- and artificially sweetened beverages) amongst Norwegian adolescents between 2016 to 2019 according to gender and SES.

## Methods

### Study design and subjects

The present study was based on two cross-sectional studies conducted in 2016 and 2019, described in detail elsewhere (24). In short, data in both studies were collected in school settings in all 30 municipalities of the county of Agder in Southern Norway. Students attending junior high school, grades 8–10 (age 13–16 years) and high school, grade 11 (age 16–17 years) were invited to participate. The first data collection was conducted during February and March 2016, and a total of 13,239 students agreed to participate, including 9,803 from junior high school (response rate 90%), and 3,436 from high school (response rate 80%). The second data collection was conducted during February and March 2019, in which a total of 12,757 students agreed to participate, including 9,547 from junior high (response rate 89%), and 3,210 from high school (response rate 81%). The participants were overall representative for the population with regard to school organisation (public or independent). Background characteristics of the participants indicated somewhat higher parental educational level compared to national registers (25).

Both in 2016 and 2019, the participants spent approximately 30 min to complete an online self-administered frequency questionnaire at school, using identical procedures at both time points of the data collection. All students provided informed consent before study participation. Both students and parents were given oral and written information about the study; and the parents could withdraw their children from participating at any time. Amongst junior high school students, all data were collected anonymously, whereas personal data was obtained for high school students. The study was approved by The Faculty Ethical Committee at The University of Agder, and the Norwegian Centre for Research Data (NSD) approved the study protocol used in high school settings. The study was conducted in line with the Declaration of Helsinki.

### Measures

#### Dietary habits

Consumption of foods and beverages was assessed by asking respondents how often they eat vegetables, fruit and berries, whole-grain bread, fish, salty snacks, and sweets, and how often they drink sugar-sweetened beverages and artificially sweetened beverages. Identical questionnaires were completed by students in junior high school and students in high school. The wording and sequence of the questions were identical in 2016 and 2019, but the questionnaires had slightly different number of response alternatives for consumption frequency. In both questionnaires, the three lowest frequency categories were ‘never’, ‘less than one time/week’, and ‘one time/week’; and the two highest frequency categories ‘every day’ and ‘several times/day’ were identical. In total, the food and beverage questions used in 2016 and 2019 had 10 and seven frequency categories, respectively. To obtain comparable frequencies, response alternatives in 2019 were coded as follows: 1) Never (coded 0); 2) Less than one time/week (coded 0.5); 3) One time/week (coded 1); 4) Two-three times/week (coded 2.5); 5) Four-six times/week (coded 5); 6) Every day (coded 7); 7) Several times/day (coded 10). Response alternatives in 2016 on the other hand were coded as follows: 1) Never (coded 0); 2) Less than one time/week (coded 0.5); 3) One time/week (coded 1); 4) Twice a week (coded 2.5); 5) Three times/week (coded 2.5); 6) Four times/week (coded 5); 7) Five times/week (coded 5); 8) Six times/week (coded 5); 9) Every day (coded 7); 10) Several times/day (coded 10).

### Socio-demographic variables

Identical questions and response alternatives were used to obtain information on socio-demographic variables in the data collections in 2016 and 2019. Class-level was applied as a proxy for age, and was measured by asking respondents which class-level they attended. Grades 8–10 cover the age range 14–16 years, and grade 11 covers the age range 16–17 years.

Socio-economic status was assessed by two questions about maternal and paternal educational level, respectively: ‘Does your mother/father have college/university-level education’ with response alternatives ‘Yes’ and ‘No’ for each parent. Further, responses based on these two questions were collapsed and recoded into three values: 1) Low education (none of the parents had higher education); 2) Medium education (one of the parents had higher education); 3) High education (both parents had higher education).

### Statistical analysis

**Fig. 1:**
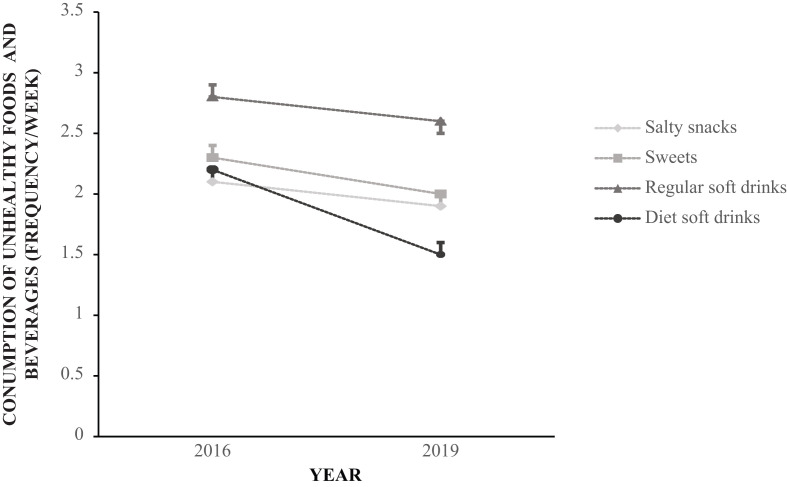
Unadjusted mean consumption frequency (times per week) of salty and sweet snacks, sugar-sweetened beverages, and artificially sweetened beverages among Norwegian adolescents in 2016 and 2019.

**Fig. 2:**
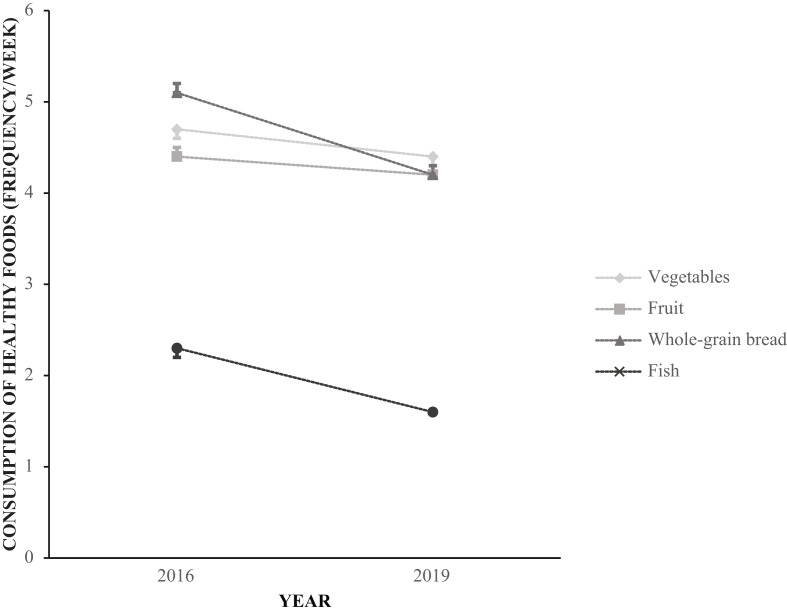
Unadjusted mean consumption frequency (times per week) of vegetables, fruit, whole-grain bread, and fish among Norwegian adolescents in 2016 and 2019.

Chi-square tests were used for comparison of gender, school level, and parental education according to study year (2019 vs. 2016). and independent samples *t*-tests were used for comparison of mean weekly frequency of consumption of foods and beverages according to study year (Figs. 1 and 2). General linear models were used to examine consumption of food and beverages in the total sample according to gender and parental education and study year (Tables 2 and 3). To examine whether time trends in food and beverage consumption varied across genders and parental education level, statistical interactions of gender and parental educational level with study year were tested (Tables 4 and 5). Results from Kolmogorov–Smirnov test indicated normally distributed data. The level of statistical significance was set to 5%. All statistical analyses were performed using Statistical Package for the Social Sciences (SPSS) Statistics 25 (IBM Corporation).

## Results

### Background characteristics

In 2016 and 2019, a total of 13,412 and 12,801 adolescents participated in the study, respectively. The distribution of participants across gender and school level was similar in 2016 and 2019, while there was a slightly higher level of parental education amongst adolescents participating in 2019 (61% vs. 57% with high parental education, *P* < 0.001) (Table 1).

**Table 1:** Background characteristics of included participants by study year

Variables	2016	2019	*P* ^1^
**Number of participants**	13,412	12,801	
**Girls *n* (%)**	6,318 (49.1)	6,308 (49.4)	0.589
**Parental education *n* (%)** ^2^			
Low	2,302 (20.2)	1,866 (17.6)	
Medium	2,627 (23.1)	2,261 (21.4)	
High	6,449 (56.7)	6,462 (61.0)	<0.001
**School level *n* (%)** ^3^			
8th grade	3,132 (24.3)	3,125 (25.4)	
9th grade	3,105 (24.1)	3,033 (24.6)	
10th grade	3,223 (25.0)	2,943 (23.9)	
11th grade	3,436 (26.6)	3,210 (26.1)	0.053

1 Differences in gender, grade and parental education were analysed using the *X^2^* test.

2 Low educated, if none of the parents had any higher education; medium educated, if one of the parents had higher education; and high educated, if both parents had higher education.

3 Grades 8–10 cover the age range 14–16 years, and grade 11 covers the age range 16–17 years.

### Time trends

The reported frequency of consumption of vegetables, fruit and berries, whole-grain bread, and fish was lower in 2019 compared with 2016 (*P* < 0.001 for all; Fig. 1 and Table 2). There was also a decreased frequency of consumption of salty snacks, sweets, sugar-sweetened beverages, and artificially sweetened beverages (*P* < 0.001 for all; Fig. 2 and Table 2).

**Table 2:** Mean and 95% confidence intervals (95% CI) weekly consumption of healthy food items among Norwegian adolescents according to gender, parental education, and study year

Variables	Total (*n*)	Vegetables (times/week)		Fruit and berries (times/week)		Whole-grain bread (times/week)		Fish (times/week)	
		Mean (95% CI)	*P* *	Mean (95% CI)	*P* *	Mean (95% CI)	*P* *	Mean (95% CI)	*P* *
**Gender**									
Boy	13,000	4.2 (4.1, 4.2)		3.8 (3.8, 3.9)		4.7 (4.7, 4.8)		2.0 (1.9, 2.0)	
Girl	12,626	4.9 (4.8, 4.9)	<0.001	4.8 (4.7, 4.9)	<0.001	4.6 (4.6, 4.7)	0.036	1.9 (1.8, 1.9)	0.001
**Parental education**									
Low	4,148	4.1 (4.0, 4.1)		3.9 (3.8, 4.0)		4.2 (4.1, 4.3)		1.8 (1.7, 1.9)	
Medium	4,888	4.4 (4.3, 4.4)		4.1 (4.1, 4.2)		4.6 (4.5, 4.7)		1.9 (1.8, 1.9)	
High	12,911	4.9 (4.8, 4.9)	<0.001	4.6 (4.6, 4.7)	<0.001	5.0 (4.9, 5.0)	<0.001	2.0 (2.0, 2.1)	<0.001
**Year**									
2016	13,418	4.7 (4.6, 4.7)		4.4 (4.4, 4.5)		5.1 (5.1, 5.2)		2.3 (2.2, 2.3)	
2019	12,801	4.4 (4.3, 4.4)	<0.001	4.2 (4.2, 4.3)	<0.001	4.2 (4.2, 4.3)	<0.001	1.6 (1.6, 1.6)	<0.001

* Differences between categories of gender, education and study year were tested using general linear models.

### Food consumption according to gender

When combining the results from the 2016 and 2019 data collections, girls reported a significantly more frequent consumption of vegetables, fruit and berries, and sweets. Boys reported a more frequent consumption of whole-grain bread, fish, salty snacks, sugar-sweetened beverages, and artificially sweetened beverages than girls (Tables 2 and 3).

**Table 3:** Mean and 95% confidence intervals (95% CI) weekly consumption of unhealthy food items among Norwegian adolescents according to gender, parental education, and study year

Variables	Total (*n*)	Salty snacks (times/week)		Sweets (times/week)		Sugar-sweetened beverages (times/week)		Artificially sweetened beverages (times/week)	
		Mean (95% CI)	*P* *	Mean (95% CI)	*P* *	Mean (95% CI)	*P* *	Mean (95% CI)	*P* *
**Gender**									
Boy	13,000	2.0 (2.0, 2.1)		2.1 (2.0, 2.1)		2.9 (2.9, 2.9)		1.9 (1.9, 1.9)	
Girl	12,626	1.9 (1.9, 2.0)	0.001	2.2 (2.2, 2.3)	<0.001	2.5 (2.4, 2.5)	<0.001	1.8 (1.7, 1.8)	<0.001
**Parental education**									
Low	4,148	2.1 (2.1, 2.2)		2.3 (2.2, 2.3)		3.1 (3.0, 3.2)		2.0 (1.9, 2.1)	
Medium	4,888	2.0 (2.0, 2.1)		2.2 (2.1, 2.2)		2.9 (2.8, 2.9)		2.0 (1.9, 2.0)	
High	12,911	1.9 (1.9, 2.0)	<0.001	2.1 (2.1, 2.1)	<0.001	2.5 (2.5, 2.5)	<0.001	1.8 (1.7, 1.8)	<0.001
**Year**									
2016	13,418	2.1 (2.1, 2.1)		2.3 (2.3, 2.4)		2.8 (2.8, 2.9)		2.2 (2.1, 2.2)	
2019	12,801	1.9 (1.9, 1.9)	<0.001	2.0 (1.9, 2.0)	<0.001	2.6 (2.5, 2.6)	<0.001	1.5 (1.5, 1.6)	<0.001

* Differences between categories of gender, education and study year were tested using general linear models.

### Food consumption according to parental education level

Compared to those with low and medium parental education, high parental education was associated with a higher frequency of consumption of vegetables, fruit and berries, whole-grain bread, and fish (Table 2). Correspondingly, there was a linear decrease in consumption frequency of salty snacks, sweets, sugar-sweetened beverages, and artificially sweetened beverages (Table 3).

### Variation in time trends across gender and parental education level

Statistically significant interactions were observed between time and gender for the frequency of consumption of fruit and berries (*P* = 0.012) and whole-grain bread (*P* = 0.005, Table 4). While consumption frequency of fruit and berries was fairly stable amongst boys between 2016 and 2019, a significant decrease in consumption frequency of −4% was seen amongst girls. A reduced frequency of consumption of whole-grain bread was observed amongst both genders over the 3-year period, but the reduction was higher amongst girls (−19%) compared to boys (−14%, Table 4). We also observed a statistically significant interaction between time and parental education on the frequency of consumption of sweets (*P* = 0.040, Table 5). Adolescents with lower and medium parental education, who reported an average higher consumption frequency of sweets in 2016 than those with high parental education, exhibited a larger decrease in consumption frequency of sweets (−17% for both) than those with high parental education (−13%). In 2019, the mean reported consumption frequency of sweets was 2 times per week regardless of parental educational level. Time trends in consumption frequency of vegetables, fish, salty snacks, and sugar- and artificially sweetened beverages did not differ significantly by gender or parental education.

**Table 4:** Mean and 95% confidence interval (95% CI) weekly consumption of healthy food items in 2016 and 2019, respectively among Norwegian adolescents according to gender and parental education

Variables	Vegetables		*P* *	Fruit and berries		*P* *	Whole-grain bread		*P* *	Fish		*P* *
	2016	2019		2016	2019		2016	2019		2016	2019	
	Mean (95% CI)	Mean (95% CI)		Mean (95% CI)	Mean (95% CI)		Mean (95% CI)	Mean (95% CI)		Mean (95% CI)	Mean (95% CI)	
**Gender**												
** Boys**	4.3 (4.2, 4.4)	4.1 (4.1, 4.2)		3.9 (3.8, 4.0)	3.9 (3.8, 4.0)		5.1 (5.1, 5.2)	4.4 (4.3, 4.5)		2.3 (2.3, 2.4)	1.7 (1.6, 1.7)	
** Girls**	5.1 (5.0, 5.2)	4.8 (4.7, 4.9)	0.056	5.0 (4.9, 5.1)	4.8 (4.7, 4.9)	0.012	5.2 (5.1, 5.3)	4.2 (4.1, 4.3)	0.005	2.2 (2.2, 2.3)	1.6 (1.6, 1.7)	0.209
**Parental education**												
** Low**	4.2 (4.0, 4.3)	3.9 (3.8, 4.1)		4.0 (3.9, 4.1)	3.8 (3.7, 3.9)		4.7 (4.6, 4.9)	3.7 (3.5, 3.8)		2.2 (2.1, 2.3)	1.4 (1.3, 1.5)	
** Medium**	4.5 (4.4, 4.6)	4.2 (4.1, 4.3)		4.2 (4.1, 4.3)	4.1 (3.9, 4.2)		5.0 (4.9, 5.1)	4.1 (4.0, 4.3)		2.2 (2.1, 2.2)	1.6 (1.5, 1.6)	
** High**	5.0 (4.9, 5.1)	4.7 (4.7, 4.8)	0.779	4.7 (4.6, 4.8)	4.6 (4.5, 4.7)	0.301	5.4 (5.3, 5.5)	4.6 (4.5, 4.7)	0.055	2.4 (2.3, 2.4)	1.7 (1.7, 1.8)	0.052

* Interactions between 1) study year x gender, and 2) study year x parental education, analysed using general linear models.

**Table 5:** Mean and 95% confidence interval (95% CI) weekly consumption of unhealthy food items in 2016 and 2019, respectively among Norwegian adolescents according to gender and parental education

Variables	Salty snacks		*P* *	Sweets		*P* *	Sugar-sweetened beverages		*P* *	Artificially sweetened beverages		*P* *
	2016	2019		2016	2019		2016	2019		2016	2019	
	Mean (95% CI)	Mean (95% CI)		Mean (95% CI)	Mean (95% CI)		Mean (95% CI)	Mean (95% CI)		Mean (95% CI)	Mean (95% CI)	
**Gender**												
** Boys**	2.1 (2.1, 2.2)	1.9 (1.9, 2.0)		2.3 (2.2, 2.3)	1.9 (1.8, 1.9)		3.1 (3.0, 3.1)	2.7 (2.7, 2.8)		2.3 (2.2, 2.3)	1.6 (1.5, 1.6)	
** Girls**	2.0 (2.0, 2.1)	1.9 (1.8, 1.9)	0.717	2.4 (2.3, 2.4)	2.1 (2.0, 2.1)	0.117	2.6 (2.5, 2.7)	2.4 (2.3, 2.4)	0.225	2.1 (2.0, 2.2)	1.5 (1.4, 1.6)	0.070
**Parental education**												
** Low**	2.2 (2.2, 2.3)	2.0 (1.9, 2.1)		2.4 (2.4, 2.5)	2.0 (2.0, 2.1)		3.2 (3.1, 3.4)	2.9 (2.8, 3.0)		2.4 (2.3, 2.5)	1.6 (1.5, 1.7)	
** Medium**	2.1 (2.0, 2.2)	1.9 (1.9, 2.0)		2.4 (2.3, 2.5)	2.0 (1.9, 2.0)		3.0 (2.9, 3.1)	2.7 (2.6, 2.8)		2.2 (2.1, 2.3)	1.6 (1.5, 1.7)	
** High**	2.0 (2.0, 2.1)	1.8 (1.8, 1.9)	0.323	2.3 (2.2, 2.3)	2.0 (1.9, 2.0)	0.040	2.6 (2.6, 2.7)	2.4 (2.3, 2.5)	0.126	2.1 (2.0, 2.2)	1.5 (1.4, 1.5)	0.064

* Interactions between 1) study year x gender, and 2) study year x parental education, analysed using general linear models.

## Discussion

Results from the present study showed a reduced frequency of consumption of selected healthy food items as well as selected unhealthy food items and beverages between 2016 and 2019. Although a general trend of reduced frequency of consumption of several healthy and unhealthy food items and beverages was observed in our study population, the reduction was most pronounced for whole-grain bread, fish, and artificially sweetened beverages. Our results also confirmed a clear socio-economic gradient in frequency of intake of all the studied food groups in favour of the adolescents with more educated parents. However, for sweets, the socio-economic gap in frequency of consumption was no longer evident in the 2019 study. The results also suggested gender differences, but the gender gap in frequency of consumption of berries and whole-grain bread was less pronounced in 2019.

As our study focused on a limited number of indicators of dietary habits, changes in consumption of other food items may partly explain the decreased consumption of all food items and beverages included in the present study. Furthermore, our findings may conceal the consequences of a general lower consumption of breakfast, irregular meals, and higher snacking amongst adolescents. Results from other studies confirm that the proportion of adolescents consuming main meals has decreased over time (26–28), and this is also supported by the current study (data not shown).

In contrast to our recently collected data, previous studies amongst North American and European, including Norwegian, adolescents indicated an overall increased consumption of fruit, berries, and vegetables between 2001 and 2010 (18, 29). Changing trends in consumption of fruit and vegetables amongst Norwegian children and adolescents during the last decades may partly be because of the implementation and termination of the nationwide free Norwegian School Fruit Scheme (NSFS), which despite being highly successful in increasing the consumption of fruit and vegetables amongst children and adolescents (30, 31) was abolished by the Norwegian government in 2014 (32). It is possible that the observed frequency of consumption of fruits, berries, and vegetables in 2016 represents a sustained effect of the NSFS, which has waned over the subsequent years. Our results also revealed a decrease in consumption of fish between 2016 and 2019. Consequently, the mean frequency of fish consumption amongst those participating in 2019 was below the national dietary recommendations of 2–3 times per week (33). Although few previous studies have reported changes in fish consumption over time, findings have confirmed a consumption of fish below national recommendations (15). The increase in consumer price of fish during the last decade (34) may partly explain the decrease in fish consumption observed in the present study. The reduced frequency of consumption of whole-grain bread, fish, fruit and vegetables amongst Norwegian adolescents suggests an overall trend of decreased consumption of selected healthy food items, which has also been confirmed by national wholesale of fish, fruit and vegetables between 2016 to 2018 (35). Thus, methods and strategies described by Norwegian National Action Plan for a Healthier Diet to increase the consumption of these healthy food items amongst Norwegian adolescents should be re-evaluated.

On the other hand, our results also indicated a positive trend towards a decreased consumption of unhealthy food items and beverages. In line with results from the present study, a decreased consumption of sweets was observed between 2001 and 2009; whereas the consumption of salty snacks seemed to increase amongst Norwegian adolescents between 1995 and 2008 (18, 36). A subsequent decrease in consumption of sugar-sweetened beverages has also been reported in the general Norwegian population from 2001 to 2016 (22). In line with our results, a study amongst Swedish adolescents also reported a reduced consumption frequency of unhealthy food items, such as sweets and salty snacks, but the authors highlighted the importance of public health actions to further improve dietary habits amongst adolescents (37). The significant increase in taxes on sugar-sweetened and artificially sweetened foods and beverages, introduced by the Norwegian government in early 2018 (38), may partly have influenced the consumption of such beverages. Several studies have found that increased taxes on unhealthy food items may effectively and positively impact dietary habits, and consequently population’s health (39–41).

The present study indicated gender differences in consumption of all included food and beverages. The gender differences were not consistently related to healthy or unhealthy dietary habits. Girls consumed vegetables, fruits and berries more often than boys, whereas boys reported a more frequent consumption of whole-grain bread and fish. Further, girls consumed sweets more often, whereas boys consumed salty snacks and sugar- and artificially sweetened beverages more often than girls. In addition, a larger decrease in frequency of consumption of fruit and berries and whole-grain bread was observed amongst girls compared to boys between 2016 and 2019, indicating a reduced gender gap in consumption of these specific food items. Previous studies amongst European adolescents have confirmed that boys tend to eat more unhealthy diets characterised by lower intake of fruit and vegetables and higher intake of sugar-sweetened beverages, than girls (16, 37, 42). These gender differences in dietary habits have partly been explained by factors such as more positive attitudes to healthy eating, increased nutrition knowledge and food motivation, and more concern about body image amongst girls compared to boys (43, 44). In the randomised controlled Special Turku Coronary Risk Factor Intervention Project study, however, a more pronounced positive effect of 20-year infancy-onset dietary intervention on food consumption and nutrient intake was found amongst boys than girls (45). This highlights the importance of developing targeted dietary interventions to decrease gender differences in dietary habits.

In line with our results, other studies have also shown an overall pattern of healthier dietary habits amongst those with higher SES (17, 21, 22, 46, 47). The proportion of participants reporting high parental education was slightly higher in 2019 compared to those participating in 2016. This change in educational level is in line with national data showing a general trend of increasing education level in the population of Norway: the number of women with up to 4 years of college/university education increased by 1.3 percentage points, and the number of women with more than 4 years of college/university education increased by 1.1 percentage points from 2016 to 2019 (48). As a result of changes in educational level over time, we might have expected results showing both increased consumption of healthy food items and decreased consumption of unhealthy food items between 2016 and 2019. However, in our data, a varying time trend by parental education level was seen only for consumption of sweets, and the socioeconomic gap in frequency of consumption of sweets seemed to disappear during this time period.

A major strength of the present study is that it comprises two cross-sectional studies with a high participation rate (between 80 and 90% depending on grade level) in a well-defined population which is expected to be representative for Norwegian adolescents with regard to socioeconomic background and school organisation (public or independent). Our study included a high number of participants and provided information about consumption of a selection of important foods and beverages amongst adolescents from the same region in 2016 and 2019. When comparing the two time points, seasonal variation in dietary consumption has had a negligible impact on the results as both data collections were carried out in February and March. Another strength is that our results may be applied in the evaluation process of the Norwegian National Action Plan for a Healthier Diet which specifically focused on increasing the frequency of healthy food items amongst adolescents between 2017 and 2021 (extended to 2023).

Our research also had limitations. We used data collected from only one (Agder) out of 11 Norwegian counties. We cannot exclude the possibility that the dietary patterns observed in Agder differ from the diet in other parts of Norway. However, according to results from other studies examining regional differences in dietary patterns, dietary patterns in Agder seem to be largely in line with those in Norway as a whole (14, 49). Furthermore, we cannot rule out that differences in number of response alternatives used in otherwise identical questionnaires, may have affected the results. When interpreting time trends in dietary intake, slight modifications in the methods over time are common and must be taken into consideration (50). In our study, the food and beverage questions used in 2016 and 2019 had 10 and seven frequency categories, respectively. For the three lowest and the two highest response alternatives, the frequencies were identical at both time points, while values had been collapsed for the middle alternatives (2016: 2/week, 3/week, 4/week, 5/week, 6/week; 2019: 2–3/week, 4–6/week). This enabled us to recode the variables into common frequencies without any appreciable loss of information. Additional analyses applying the original 2016 frequency categories (data not shown) indicate that these minor differences in response alternatives did not influence our conclusions. Another limitation is that the food frequency questionnaire used in the present study did not provide information about portion sizes and quantity of food and beverages consumed. Finally, as our study only provided information about frequency of consumption of selected foods and beverages, we have no information about changes in consumption of other food groups which may help explain our findings.

## Conclusion

Our results indicate a reduced consumption of both healthy food items and unhealthy food items, and beverages amongst adolescents between 2016 and 2019. Despite some differences in frequency of food and beverage consumption amongst boys and girls and a general trend indicating healthier dietary habits amongst adolescents with higher parental education, the gender gap in consumption of fruit and berries and whole-grain bread seemed to decrease, and the socioeconomic gap in consumption of sweets seemed to be closed during this time period. Thus, positive trends in consumption of diet and beverage have been identified, but future efforts should specifically focus on increasing the consumption of healthy food items and develop targeted intervention strategies to further decrease the gender and socio-economic gap in dietary habits.
